# Cross-sectional comparison of critically ill pediatric patients across hospitals with various levels of pediatric care

**DOI:** 10.1186/s13104-015-1550-9

**Published:** 2015-11-19

**Authors:** Brian D. Benneyworth, William E. Bennett, Aaron E. Carroll

**Affiliations:** Section of Pediatric Critical Care Medicine, Department of Pediatrics, Indiana University School of Medicine, 705 Riley Hospital Dive, RI 2117, Indianapolis, IN 46202 USA; Section of Pediatric Gastroenterology, Department of Pediatrics, Indiana University School of Medicine, 705 Riley Hospital Dive, RI 4210, Indianapolis, IN 46202 USA; Department of Pediatrics, Center for Pediatric and Adolescent Comparative Effectiveness Research, Indiana University School of Medicine, 410 West 10th Street, HS Suite 4099C, Indianapolis, IN 46202 USA

**Keywords:** Pediatric Health Information System, Kids’ Inpatient Database, Pediatric critical care, Intensive care

## Abstract

**Background:**

Inpatient administrative data sources describe the care provided to hospitalized children. The Kids’ Inpatient Database (KID) provides nationally representative estimates, while the Pediatric Health Information System (PHIS, a consortium of pediatric facilities) derives more detailed information from revenue codes. The objective was to contextualize a diagnosis and procedure-based definition of critical illness to a revenue-based definition; then compare it across hospitals with different levels of pediatric care.

**Methods:**

This retrospective, cross-sectional study utilized the 2009 KID, and 2009 inpatient discharges from the PHIS database. Patients <21 years of age (excluding neonates) were included to focus on pediatric critical illness. Critical illness was defined as: (1) critical care services (CC services) using diagnosis and procedures codes and (2) intensive care unit (ICU) care using revenue codes. Demographics, invasive procedures, and categories of critical illness were compared using Chi square and survey-weighted methods. The definitions of critical illness were compared in PHIS hospitals. CC services populations identified in General Hospitals, Pediatric Facilities, and Freestanding Children’s hospitals (from KID) were compared to those in PHIS hospitals.

**Results:**

Among PHIS hospitals, critically ill discharges identified by CC services accounted for 37.7 % of ICU care. CC services discharges were younger and had greater proportion of respiratory illness and invasive procedure use. Critically ill patients identified by CC services in PHIS hospitals were statistically similar to those in Freestanding Children’s hospitals. Pediatric Facilities and General Hospitals had more adolescents with more traumas. CC services patients in general hospitals had lower use of invasive procedures and predominance of trauma, respiratory illness, mental health issues, and general infections. Freestanding children’s hospitals discharged 22 % of the estimated 96,700 CC services cases. Similar proportions of critically ill patients were seen in Pediatric Facilities (31 %) and General Hospitals (33 %).

**Conclusion:**

The CC services definition captured a more severely ill fraction of critically ill children. Critically ill discharges from PHIS hospitals can likely be extrapolated to Freestanding Children’s hospitals and Pediatric Facilities. General Hospitals, which provide a significant amount of pediatric critical care, are different. Studies utilizing administrative data can benefit from multiple data sources, which balance the individual strengths and weaknesses.

**Electronic supplementary material:**

The online version of this article (doi:10.1186/s13104-015-1550-9) contains supplementary material, which is available to authorized users.

## Background

Pediatric critical illness (outside of the neonatal period) is often unexpected and is accompanied by rapid deterioration so children present to the closest hospital for care. Injury, infections, and respiratory disease (including asthma/bronchiolitis) are among the most common reasons for hospital admission and can require critical care level services [1]. Hospitals provide varying levels of pediatric care including general hospitals without designated pediatric rooms, a dedicated pediatric unit/floor, a designated pediatric hospital within a larger adults system, and a complete freestanding pediatric hospital.

While critically ill adults have been described with the Medicare administrative database or Veterans Affairs electronic medical record [2], descriptions of critically ill children have relied on several administrative data sources each with their own strengths and weaknesses: (1) Kids’ Inpatient Database (KID), (2) the Pediatric Health Information System (PHIS), (3) the National Children’s Hospitals and Related Institutions (NACHRI) Case-Mix database, and (4) various state Medicaid databases. The KID and PHIS databases are the largest and most utilized. Administrative data sources abstract information from the universal billing form, which contains patient demographics, diagnoses, procedures, revenue codes, and diagnosis related groups (DRG). While subject to biases and inaccuracies [3–5], these data sources provide standardized information on large numbers of patients across numerous hospitals.

The KID is the largest of these administrative data sources including discharges from 4121 hospitals (representing the entire spectrum of levels in pediatric care) from 44 states [6]. It is compiled from the State Inpatient Databases. It utilizes a specific sampling frame to permit nationally representative estimations of the inpatient care provided to children. It contains basic demographic, diagnosis, procedure, and DRG codes but does not contain revenue codes for specific services because of differences in data sharing agreements at the state level. Descriptions of critically ill populations have therefore relied on combinations of diagnosis and procedure codes to identify illnesses that were likely managed in the pediatric intensive care unit (PICU) [7]. Because KID is nationally representative, it has been useful in defining incidence of admission and overall healthcare utilization over time [8–10].

PHIS aggregates non-sampled administrative data from 44 pediatric hospitals (a subset of the approximately 250 Children’s Hospital Association members who all provide dedicated pediatric care). Because of a collaborative agreement, revenue codes are included this administrative dataset. These revenue codes can identify specific services from intensive care units or those services related to PICU level therapies (e.g. mechanical ventilation). These revenue codes permit additional analysis of critically ill patients but only on a subset of hospitals [11, 12].

No single pediatric dataset can provide a nationally representative view of pediatric critical care across hospitals with varying levels of pediatric care. This study hypothesizes that critically ill children are cared for in hospitals with all levels of pediatric care and that defining critical care based only on diagnosis and procedure codes underestimates the true incidence of pediatric critical care. The objectives were to: (1) compare the populations described by two definitions of critical care (revenue codes versus diagnosis and procedure codes) and (2) compare critically ill patients (defined by diagnosis and procedure codes) across hospitals with different levels of pediatric care.

## Methods

### Study design and datasets

This was a retrospective, cross-sectional study evaluating pediatric discharges for critically ill patients in 2009. This study compared a definition of critical illness using diagnosis and procedure codes in the context of a revenue codes definition. Then it utilized two different datasets (KID and PHIS) in order to facilitate comparisons across hospitals that provide various levels of pediatric care. This study was approved by the Institutional Review Board of Indiana University.

The 2009 KID is compiled by the Agency for Healthcare Research and Quality’s (AHRQ) Healthcare Cost and Utilization Project (HCUP) and available for purchase from the HCUP Central Distributor. KID is a nationally representative database that samples 80 % of pediatric discharges and 10 % of uncomplicated births in order to increase the power to detect and evaluate rare conditions. Discharges are weighted based on the sampling scheme to permit inferences for a nationally representative population. In 2009, the KID contained de-identified information on approximately 7.4 million weighted discharges from 4121 hospitals in 44 states. HCUP provides further sampling and weighting details. The dataset contains demographic information as well as International Classification of Disease, Version 9, Clinical Modification (ICD-9-CM) diagnosis and procedural coding [6]. KID identifies hospitals using the NACHRI designations: (1) General hospital without pediatric facilities, (2) Children’s general care hospital (freestanding), (3) Children’s specialty hospital, and (4) dedicated pediatric facilities in a general hospital. Because of the small number of discharges and care is usually focused on specific diagnoses (e.g. burn facilities) from hospitals with the designation of children’s specialty hospital, those hospitals were excluded.

PHIS is an administrative database that contains inpatient, emergency department, and ambulatory surgery data from 44 non-for-profit, tertiary care pediatric hospitals with teaching services in the United States. The hospitals are affiliated with the Children’s Hospital Association (CHA; Shawnee Mission, KS), a business alliance of children’s hospitals and is freely available to member hospitals. Data quality and reliability are assured through a joint effort between the CHA and participating hospitals. The data warehouse function for the PHIS database is managed by Thomson Reuters (Ann Arbor, MI, USA). Data are de-identified at the time of submission, and are subjected to a number of reliability and validity checks before being included in the database. All discharges at each hospital are included. Similar demographic and diagnosis/procedural information to that in the KID are included, but PHIS also has revenue codes mapped to clinical transaction classification (CTC) system, which permits more detailed evaluation of billed services during a hospitalization [11, 12]. Two hospitals that do not submit revenue codes were excluded from analysis. PHIS hospitals are a combination of freestanding children’s hospitals and large pediatric hospital within academic health systems, which are separate NACHRI designations in KID.

### Identification of sample

In order to compare critical illness in these datasets, two definitions of critical illness were used. ICU care was defined using the CTC revenue codes, found only in PHIS, which reflect a nursing unit charge for intensive care. As with all revenue codes, an actual PICU admission cannot be verified and this definition can only be used in large children’s hospitals that submit data to PHIS. This definition has been used to define pediatric critical illness [13–15], but non-PHIS children’s hospitals and hospitals with more limited pediatric care are not represented. In order to evaluate critical illness across a broader range of pediatric facilities a non-revenue code definition, critical care services (CC services), was defined using the primary or secondary ICD-9-CM diagnostic or procedure codes. CC services were defined by the presence of an ICD-9-CM code for cardiac or pulmonary arrest (799.1), respiratory failure (518.8×), apnea (786.03), or delivery of invasive mechanical ventilation (96.7×) [7]. The CC services set of coding underrepresents critical illness, as it does not include illness with varying degrees of severity such as respiratory distress, hemodynamic instability, neurological changes, or post-operative monitoring. This definition was chosen in order to identify discharges with the highest probability of requiring critical care [7, 16–18]. The CC services was applied in both datasets (KID and PHIS) because ICD-9-CM codes are common to each.

This analysis was focused on identifying pediatric critical illness. Neonatal and newborn care was excluded using ICD-9-CM diagnosis codes and neonatal All Patient Refined Diagnostic Related Groups (APR DRGs) [10]. The complete list is delineated in Additional file 1, but encompasses routine and premature newborn care. KID discharges are limited to less than 21 years of age so PHIS discharges were similarly limited.

### Sample characteristics

Demographic variables from both datasets were included in the analysis. Age was defined in whole years and treated as a categorical variable because of its non-normal distribution. Race/ethnicity was evaluated in the following categories: (1) White, Non-Hispanic; (2) African American; (3) Hispanic; (4) Other; and (5) missing (approximately 20 % of KID and 15 % of PHIS discharges are missing). Primary payers were grouped into public sources (Medicaid and Medicare), private sources, and other types (including self-pay, no charge, and other sources).

The incidence of invasive procedures among both critically ill populations was identified using primary and secondary ICD-9-CM procedure codes. Acute mechanical ventilation was identified by 96.7×. Non-Invasive mechanical ventilation was 93.9×. Arterial Catheterization was either 38.91 or 89.61. Central venous line catheterization was 38.92–95 or 89.62–64. Blood product transfusion was identified by 99.0×.

To evaluate the primary type of critical care provided during the hospitalization, the assigned APR DRG was grouped into the following categories of critical illness: (1) respiratory disease; (2) surgical procedures; (3) Trauma and head injury; (4) seizures and neurologic diagnoses; (5) Cardiac; (6) ingestion/toxin exposure/mental health; (7) general infections/sepsis; (8) ECMO (Extracorporeal membrane oxygenation) or tracheostomy; (9) any hematology or oncology; and (10) other diagnoses. A complete list of APR DRG mapping is available in Additional file 1. Chronic complex conditions (CCC) were identified among the ICD-9-CM diagnosis codes [19].

### Data analysis

#### Comparison of CC services and ICU care definitions of critical care

In order to evaluate the proportion of discharges captured by the CC services definition as compared to the ICU care definition these populations were compared in the PHIS database. Demographics, invasive procedures, and critical illness category were compared using Pearson’s χ^2^.

#### Comparisons between CC services populations among hospital types

Critically ill populations, as defined by the CC services definition, were compared between PHIS hospital discharges and hospitals with various levels of pediatric care as defined by the NACHRI definition in KID: (1) General hospitals (without dedicated pediatric facilities), (2) Pediatric facilities (as part of a general hospital), and (3) Freestanding children’s hospitals. Pediatric facilities included hospitals with small pediatric floors and stand-alone children’s hospitals that are part of an adult system which are not freestanding. PHIS hospital discharges were merged with the KID in order to allow the comparison. These comparisons were performed to permit: (1) extrapolation of the CC services versus ICU care definitions of critical care to all hospitals and (2) comparison of the mixed hospital type (freestanding and larger pediatric facilities) that make up the PHIS hospital network to other levels of pediatric care. Descriptive comparisons were made using appropriate survey-weighted methodology using Pearson’s χ^2^. The number of discharges estimated from KID was based on HCUP’s sampling methodology.

## Results

### Comparison between the definitions of critical illness utilizing the PHIS dataset

In 2009, 68,834 discharges received ICU care (as defined by revenue codes) among the 42 PHIS hospitals. PHIS hospitals all care for large numbers of critically ill patients each year with a minimum of 560 discharges and a median of 1675 discharges receiving ICU care. Of these discharges 25,954 (37.7 %) met the definition of CC services (based on ICD-9-CM diagnosis and procedure codes).

**Fig. 1:**
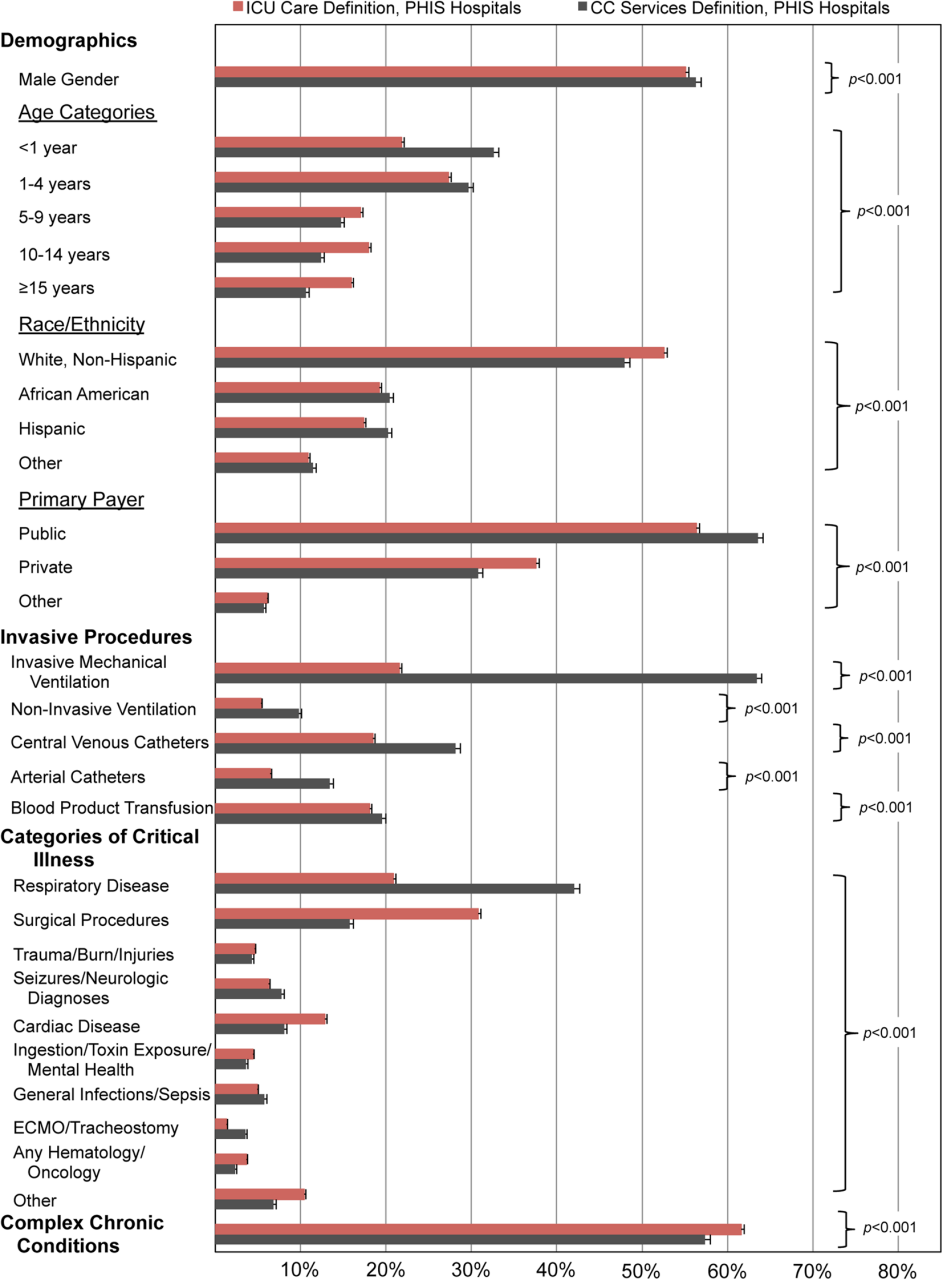
Comparison of critically ill populations defined by ICU care and CC services utilizing PHIS. Population characteristics (Demographics, Invasive Procedures, and Categories of Critical Illness) are compared to contextualize the CC services (ICD-9-CM based) definition of critical illness among discharges identified by ICU care (revenue code based definition). Proportions are delineated with the upper 95 % confidence interval for each group. Statistical comparisons are shown with respective *p* values

Critical illness discharges defined by ICU care and CC services represent distinct populations (Fig. 1). The CC services definition captures a population whose age distribution is skewed towards infants and young children. There are small statistically significant differences in the distributions of gender, race/ethnicity, and primary payers that are clinically insignificant. Given the inclusion of the mechanical ventilation as part of the definition of CC services, it is not surprising these critically ill patients have higher proportions of invasive procedures. In comparing the categories of critical illness, the CC services definition captures a higher proportion of respiratory infections with few discharges related to surgical care and cardiac disease.

### Critical illness (CC services) discharges among different hospital types

In 2009, there were an estimated 3.1 million pediatric discharges in the US (excluding discharges likely resulting from neonatal and/or NICU care). Of these discharges, 56.3 % occurred in general hospitals without dedicated pediatric services, 20.2 % occurred in one of 205 pediatric facilities, and 12.6 % occurred in one of the 32 freestanding children’s hospitals. The 42 PHIS hospitals had 452,776 discharges (approximately 15 % of pediatric discharges nationwide).

**Fig. 2:**
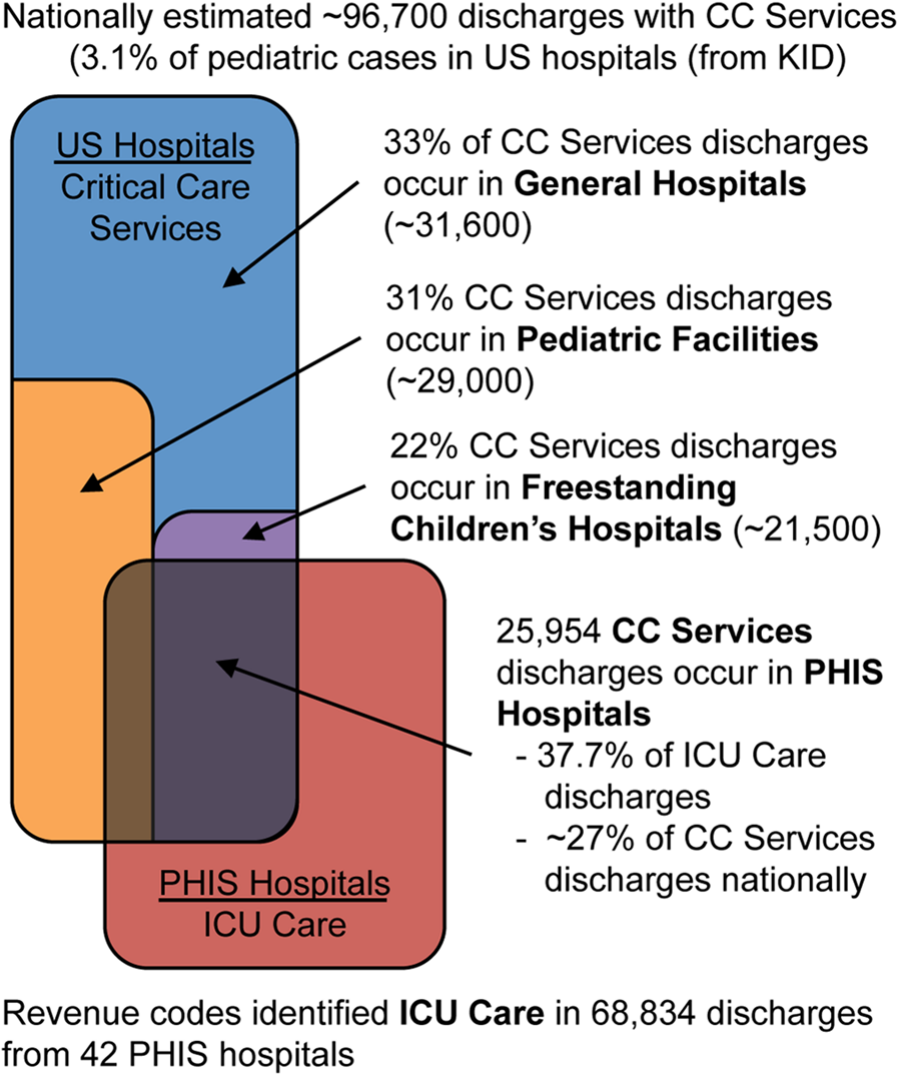
Distribution of pediatric critical illness across hospital types. Nationally representative estimates (from KID) for critically ill discharges as defined by CC services among Freestanding Children’s Hospitals, Pediatric Facilities within general hospitals, and General Hospitals without dedicated pediatric facilities are shown. Estimated overlap of CC services discharges from PHIS hospitals as a proportion of the discharges for ICU care in PHIS hospitals is shown. *Colors* coding is consistent with population characteristics in Fig. 3

Utilizing the CC services definition, there was an estimated 96,700 critically ill pediatric discharges in the US (3.1 % of all pediatric discharges). As indicated in Fig. 2, these discharges for critical illness are distributed among all types of hospitals, [e.g. General hospitals without pediatric facilities (33 %), Pediatric facilities (31 %), and Freestanding children’s hospitals (22 %)]. With 25,954 discharges meeting the CC services definition, PHIS hospitals only account for 27 % of these critically ill discharges.

**Fig. 3:**
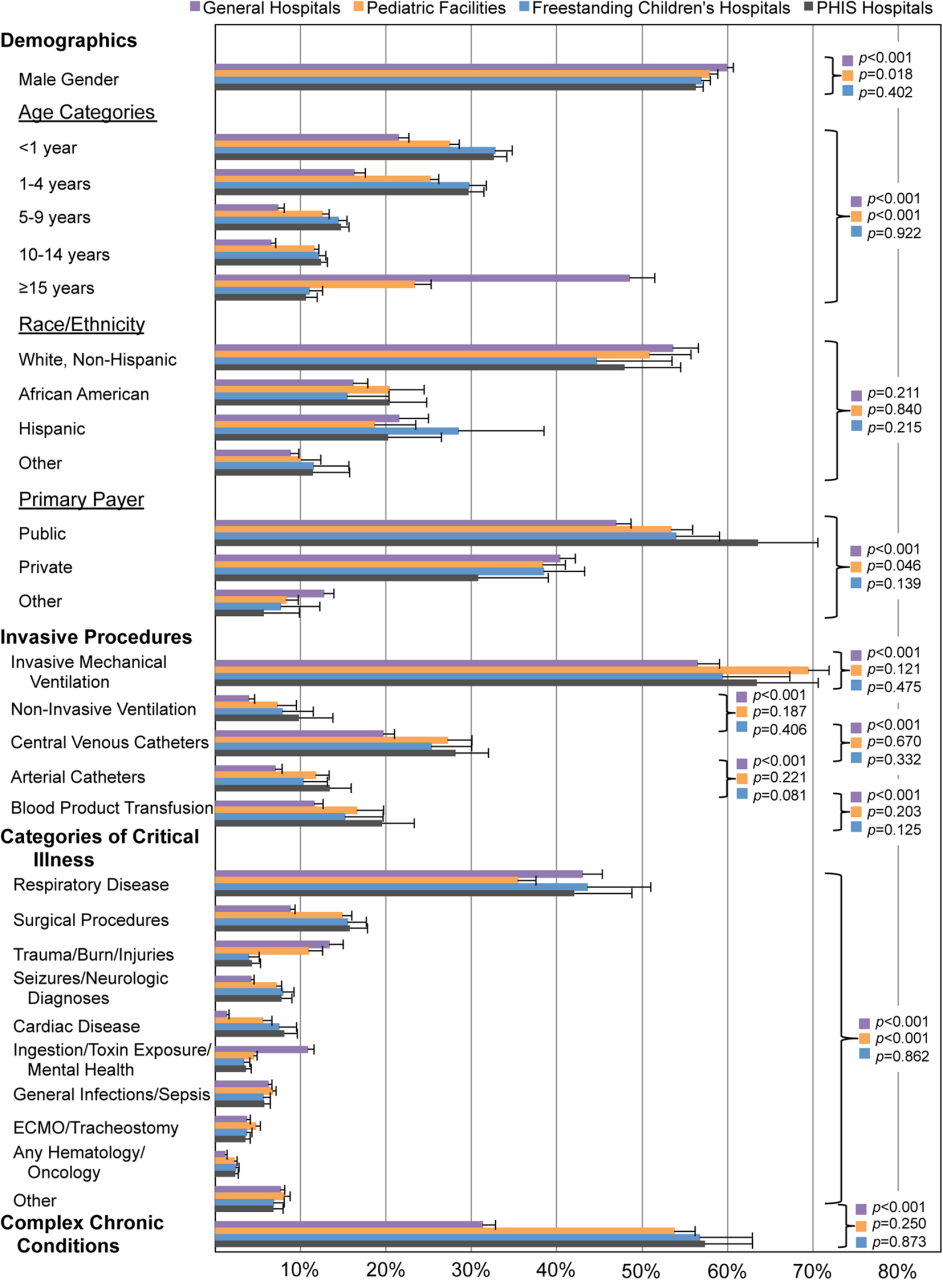
Comparison of populations of critically ill patients defined by CC services across hospitals with different levels of pediatric care. Population characteristics (Demographics, Invasive Procedures, and Categories of Critical Illness) are compared to identify differences in the CC services definition of critical illness among PHIS hospitals, Freestanding Children’s Hospitals, Pediatric Facilities within general hospitals, and General Hospitals without dedicated pediatric facilities. Proportions are delineated with the upper 95 % confidence interval for each group. Statistical comparisons between each hospital type and PHIS hospitals are shown with respective *p* values

Critical illness discharges in Freestanding children’s hospitals and PHIS hospitals are largely comparable and likely have considerable overlap as many freestanding hospitals are PHIS members. PHIS hospitals all had at least 200 CC services related discharges, as did freestanding children’s hospitals. Proportional characteristics of demographics, invasive procedures, and categories of critical illness are statistically similar between PHIS hospitals and Freestanding children’s hospitals (Fig. 3).

Pediatric Facilities within general hospitals that care for critically ill children represent a broad group of hospitals. Among this hospital cohort of 108 hospitals, 63 had more than 200 CC services related discharges and account for 80 % of the estimated 29,900 discharges in this group. This critically ill population is similar to the PHIS hospitals but is skewed toward older adolescents ages 16–20 years. There are likely clinically insignificant differences in gender and primary payer. The proportions of invasive procedures are similar, but there are higher proportions of trauma with less cardiac and respiratory disease (Fig. 3).

General hospitals, as expected, are a substantially different group of hospitals than PHIS hospitals. There are 2221 facilities each contributing a median of 5 discharges (interquartile range 3–11) related to CC services. Discharges from general hospitals are substantially older with 48.5 % of patient ≥15 years of age. While gender and ethnicity are similar, there are a greater proportion of private payers. There is less use of invasive procedures and discharges for chronic illness. Differences in the categories of critical illness are likely driven by greater trauma and toxin exposure/mental health and less of the other categories except general infections and respiratory illness (Fig. 3).

## Discussion

To our knowledge this is the first study to identify differences in the pediatric critically ill patients across various types hospitals that provide different levels of pediatric care. Prior work with administrative data sources have focused on clinical entities, like sepsis and asthma, which may be cared for in the PICU [20–24]. Procedures common to critically ill populations like mechanical ventilation, tracheostomy, and intracranial pressure monitoring have also been described [10, 25–29]. Revenue codes in the PHIS database have been used to define practice variation in diseases like asthma, bronchiolitis, and empyema [13–15]. This study contextualized the diagnosis and procedure code definition of critical illness (CC services) with a revenue code definition (ICU care) in the PHIS database. The CC services definition was then applied across various hospital types from general hospitals with no dedicated pediatric facilities to freestanding children’s hospitals. Despite the tendency to care for critically ill children in centralized hospitals with dedicated pediatric services [30, 31], 33 % of the 96,700 critically ill inpatient discharges occur in hospitals with out pediatric units (Fig. 3). This number is likely higher considering that CC services only identified 37.7 % of discharges with revenue codes for ICU care.

The CC services definition does not identify all critical illness because the definition relies on identifying cardio-pulmonary organ failure. This study confirmed this by comparing it to a definition of critical illness based on revenue codes (ICU care) in the PHIS database. As would be expected this CC services definition identified respiratory illness and patients who received a higher proportion of invasive procedures. The ICU care definition was not validated to a known population of patients who were admitted to the PICU and little is published regarding the epidemiology of pediatric critical care delivery. A PICU population with approximately 20 % respiratory infections, 30 % post-op surgical admissions, and with 20 % of all patients receiving invasive mechanical ventilation is generally appropriate for a large unit [31].

There are differences in the populations of critically ill children cared in various types of hospitals. Not surprising, the care provided in general hospitals is directed towards older adolescents, likely by providers with less pediatric experience and specialization. This trend in caring for older children is seen in general hospitals with dedicated pediatric facilities as compared to freestanding children’s hospitals. The predominant categories of critical illness in general hospitals reflect the common disease processes that require urgent care including: respiratory illness (asthma/bronchiolitis), trauma, infections, and mental health/ingestions. These are relatively common illnesses that are usually described and evaluated in hospitals with dedicated pediatric facilities.

Because of the clinical detail identified by revenue codes in PHIS, it is commonly used to evaluate pediatric care. It is unknown how the care in PHIS hospitals applies to hospitalized children in facilities with less dedicated pediatric resources. Bratton et al. in 2012 linked the PHIS database to a clinical research database, the Collaborative Pediatric Critical Care Research Network, and found that the research network hospitals were similar to the broader care provided in PHIS children’s hospitals for the treatment of status asthmaticus [11]. The critically ill populations defined by CC services are clinically similar between PHIS hospitals, freestanding children’s hospitals, and pediatric facilities in general hospitals. Among general hospitals without pediatric facilities, 43 % of discharges related to CC services had a respiratory concern (including asthma) and it is unclear how pediatric research applies to these admissions.

This analysis is limited by the retrospective use of administrative data sources including the use of ICD-9-CM and revenue codes to define the care provided to patients. The ICU revenue code used for the analysis of PHIS discharges is based on nursing unit designations that may vary from hospital to hospital in terms of what constitutes a PICU encounter. This analysis was focused on the delivery of care from the perspective of the health care system and so the readmissions for the same patient were not accounted for. Both the 2009 KID and the PHIS can account for readmissions of a patient to the same hospital, but because KID discharges are sampled there is no guarantee that a readmission would be detected. Additionally this analysis did not evaluate the effect of transfers between small hospitals and larger referral hospitals.

Overall, this analysis leverages the strengths of two administrative databases and provides a more complete picture of pediatric critical care. KID provides a nationally representative sample of all hospitalized pediatric care across a variety of institutions; it does not have enough detail to adequately characterize critical illness. PHIS can more closely define critical illness through revenue codes but does not represent all hospitals, especially those without dedicated pediatric facilities. This model of using different administrative data sources to better define and compare respective populations can be used with future studies that seek to describe other populations or disease entities which should improve the generalizability of their findings.

## Conclusion

Critically ill children represent a small but substantial portion of the hospitalized pediatric population. While much of this care is delivered in pediatric facilities, a substantial proportion is delivered in general hospitals without pediatric specific services. Results obtained from studies on PICU populations in PHIS hospitals are generalizable to freestanding children’s hospitals and potentially large pediatric units in general hospitals.

## Additional file

10.1186/s13104-015-1550-9 List of individual APR DRGs in each Category of Critical Illness.

## Abbreviations

KID: Kids’ Inpatient Database
PHIS: Pediatric Health Information System
NACHRI: National Children’s Hospitals and Related Institutions
PICU: pediatric intensive care unit
CC Services: critical care services
CTC: clinical transaction classification
AHRQ: Agency for Healthcare Research and Quality
HCUP: Healthcare Cost and Utilization Project
ICD-9-CM: International Classification of Disease, 9th Revision Clinical Modification
CHA: Children’s Hospital Association
APR DRG: All Patient Refined Diagnostic Related Groups
ECMO: extracorporeal membrane oxygenation
CCC: chronic complex conditions
