# Supplementary material for: Cross-sectional comparison of critically ill pediatric patients across hospitals with various levels of pediatric care
Source: BMC Res Notes. 2015 Nov 19;8:693. doi: 10.1186/s13104-015-1550-9 (PMC4653873; doi:10.1186/s13104-015-1550-9)
Supplement: Supplementary file 1 — 10.1186/s13104-015-1550-9 List of individual APR DRGs in each Category of Critical Illness. [file 13104_2015_1550_MOESM1_ESM.docx]

Mapping to APR DRG codes to Critical Illness categories:

Other Disease:

82, 114, 115, 134, 140, 142, 197, 199, 203, 207, 241, 242, 243, 244, 245, 246, 247, 249, 251, 252, 253, 254, 279, 280, 282, 283, 284, 340, 341, 342, 346, 347, 349, 351, 380, 381, 385, 420, 421, 422, 423, 424, 425, 460, 462, 465, 466, 468, 501, 532, 560, 561, 563, 564, 565, 566, 811, 813, 860, 861, 862, 955, 956

Respiratory infections (Upper and lower disease):

113, 130, 131, 132, 133, 137, 138, 139, 141, 143, 144

Surgical Procedures:

1, 6, 21, 22, 23, 24, 26, 70, 73, 89, 90, 91, 92, 93, 95, 97, 98, 120, 121, 169, 173, 180, 220, 221, 222, 223, 224, 225, 226, 227, 228, 229, 260, 261, 262, 263, 264, 301, 302, 303, 304, 305, 309, 310, 312, 313, 314, 315, 316, 317, 320, 321, 361, 362, 363, 364, 401, 403, 404, 405, 440, 441, 443, 444, 445, 446, 447, 480, 481, 482, 483, 484, 510, 513, 514, 517, 518, 519, 540, 541, 542, 544, 545, 546, 650, 740, 791, 850, 950, 951, 952

Trauma, Burns, and Head Injury:

20, 40, 55, 56, 57, 135, 308, 384, 711, 841, 842, 843, 844, 910, 911, 912, 930

Seizures & Neurologic diagnoses:

42, 43, 44, 45, 46, 47, 48, 52, 53, 54, 58, 111, 204

Cardiac Disease:

2, 160, 161, 162, 163, 165, 166, 167, 170, 171, 174, 175, 176, 177, 190, 191, 192, 194, 196, 198, 200, 201, 205, 206, 630

Ingestion/Toxin exposure/Mental Health:

750, 751, 752, 753, 754, 755, 756, 757, 758, 759, 760, 770, 772, 773, 774, 775, 776, 812, 815, 816

General Infections/Sepsis:

49, 50, 51, 80, 193, 248, 344, 383, 463, 531, 710, 711, 720, 721, 722, 723, 724, 890, 892, 893, 894

ECMO (Extracorporeal Membrane Oxygenation) or Tracheostomy:

4, 5

Any Hematology/Oncology:

3, 41, 110, 136, 240, 281, 343, 382, 442, 461, 500, 511, 512, 530, 651, 660, 661, 662, 663, 680, 681, 690, 691, 692, 693, 694

NICU/Neonatal Care (Exclusion category):

580, 581, 583, 588, 589, 591, 593, 602, 603, 607, 608, 609, 611, 612, 613, 614, 621, 622, 623, 625, 626, 631, 633, 634, 636, 639, 640, 863
